# Parabolic Equation Modeling of Electromagnetic Wave Propagation over Rough Sea Surfaces

**DOI:** 10.3390/s19051252

**Published:** 2019-03-12

**Authors:** Ying Gao, Qun Shao, Binzhou Yan, Qifan Li, Shuxia Guo

**Affiliations:** 1 School of Marine Science and Technology, Northwestern Polytechnical University, Xi′an 710072, China; gaoying@nwpu.edu.cn (Y.G.); ybz@mail.nwpu.edu.cn (B.Y.); liqifan@mail.nwpu.edu.cn (Q.L.); 2 Science and Technology on UAV Laboratory, Northwestern Polytechnical University, Xi′an 710072, China; guoshuxia@nwpu.edu.cn

**Keywords:** parabolic equation, electromagnetic wave propagation, rough sea surface, atmospheric dust, randomly rough sea surface model

## Abstract

The parabolic equation is an efficient numerical solution for electromagnetic wave propagation. In order to address the difficulties in predicting electromagnetic wave propagation in the maritime environment caused by atmospheric dust and rough sea surfaces, and the shortcomings of the existing research that cannot fully reflect the rough characteristics of sea surfaces, the authors have modelled electromagnetic wave propagation in the maritime environment, including in the presence of atmospheric dust. In this study the authors present a parabolic equation modeling method for calculating the electromagnetic wave propagation over rough sea surfaces. Firstly, the rough sea surface is generated by building a double summation model of three-dimensional random sea surface. Then, combined with the piecewise linear shift transformation method of the parabolic equation model, the parabolic equation random sea surface model is constructed, and the electromagnetic wave propagation characteristics in a rough sea environment are analyzed. Finally, a large number of results are compared with the Miler-Brown model and shadow effect model in rough sea environments, which verifies that the random sea surface model can better characterize the influence of rough sea surfaces on electromagnetic wave propagation. The model can be used to improve the reliability of marine microwave communication links and the detection performance of ship-borne radar.

## 1. Introduction

The electromagnetic wave propagation at sea is mainly related to the characteristics of propagation domain (atmospheric dust, etc.) and the modeling of reflection effects on the sea surface (smooth or rough surface). The sea surface is one of the important transmission ways in the process of electromagnetic wave propagation, and the sea surface environment is prone to change [1]. Therefore, it is also very difficult to predict the characteristics of electromagnetic wave propagation. The high wind waves on the sea usually have an important impact on the wireless communication system on the sea surface, especially for antennas or radar with relatively low position (such as sea-based antenna, ship-borne radar and so on). Atmospheric dust is one of the most important factors in the study of electromagnetic wave propagation characteristics in the sea environment [2]. Atmospheric dust can cause sudden changes in refractive index, and the propagation of electromagnetic waves is strongly affected (electromagnetic waves propagate along the coast) [3].

Since Leontovich and Fock deduced the parabolic equation (PE) from the Helmholtz wave equation and established the theoretical framework of tropospheric wave propagation modeling, the parabolic equation model has been showing its strong adaptability in the field of wave propagation research, which can effectively deal with complex terrain and inhomogeneous media environments [4]. After adopting the Split Step Fourier Transform (SSFT) algorithm [5], it also has the characteristics of fast calculation speed and high accuracy, so it is widely used in the prediction of radio wave propagation characteristics in large-scale environments. 

**Figure 1:**
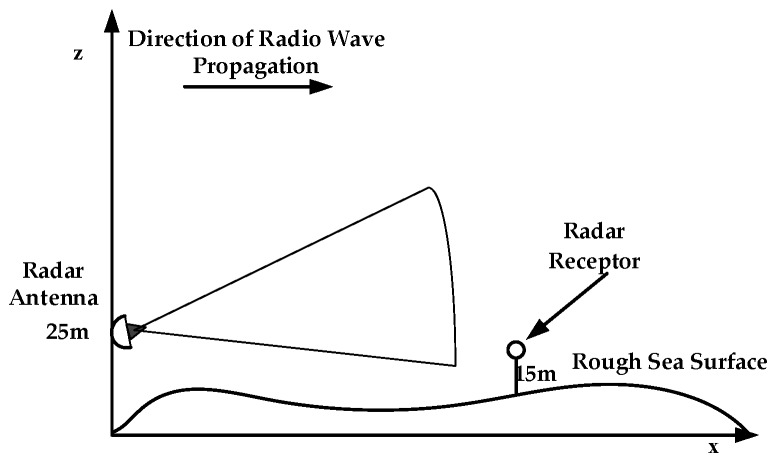
Conceptual model of a rough sea surface. The radar antenna is placed at a certain height in space, and the radar receptor is arranged in the *X* or *Z* direction in order to obtain the propagation loss on the location. Here we take the altitude of 0 m as the sea level. The radar antenna height is 25 m. The height of radar receptor is 15 m and the horizontal distance is 13 km. The sea surface roughness is related to the sea surface wind speed, where the wind speed is 7 m/s.

At present, many scholars have studied the propagation of radio waves in the marine environment based on the PE model. For example, Levy, proposed a modeling method for rough sea surfaces based on two-dimensional PE [6]. Guo from Sun Yat-sen University, analyzed the propagation characteristics of radio waves on the rough sea surface [7]; Guo from Xidian University, studied wave propagation in evaporation dusts, etc. [8]. As shown in Figure 1, different methods are used to deal with the problem of radio propagation when considering the roughness of the sea surface. The Miller-Brown method is usually used to approximate the roughness of the sea surface [9]. It approximates the roughness of the sea surface to a smooth plane, and then a roughness correction factor is used to modify the reflection coefficient of the sea surface by taking into account the influence of the roughness of the sea surface. Because the Miller-Brown model is closer to the measured data, it is generally used in engineering applications to calculate the equivalent reflection coefficient of rough sea surfaces. However, neither the Ament model nor the Miller-Brown method consider the shadow effect caused by sea waves at small grazing angles. Brokelman and Hagfors used a Monte-Carlo method to generate rough sea surfaces to solve this problem, but the computational efficiency is low [10]. Fabbro proposed a roughness attenuation factor method considering shadow effects based on the Smith method, Wagner method and Monte-Carlo method [11]. Some conclusions are given in the research of electromagnetic wave propagation under rough sea surface conditions in an ideal atmospheric environment, but all of them are approximate models. The electromagnetic scattering factors of the sea surface are not taken into account, which cannot fully reflect the shortcomings of rough sea surface characteristics. Based on the three-dimensional parabolic equation, Zhang introduced the dynamic fractal method to improve the traditional parabolic equation model, but the model analysis is insufficient [12]. Benhmammouch presented a new method based on sea surface generation using sea spectra developed to model the sea surface roughness effects on electromagnetic wave propagation, but the influence of radar emitter frequency and antenna height on radio wave propagation under rough sea surface conditions has not been studied [13].

Based on the above conclusions, the effects of atmospheric dust and rough sea surface on the propagation of radio waves at sea are analyzed in this paper. We mainly assume that the rough sea surface is a random distribution. By establishing a three-dimensional random sea surface double superposition model, we obtain the three-dimensional random sea surface wave height distribution. Combining this with the piecewise linear shift transformation method of the parabolic equation, the random sea surface model of the parabolic equation is established. Finally, we compare and analyze the influence of rough sea surfaces on radio wave propagation, and verify the efficiency and accuracy of the model.

## 2. Parabolic Equation Method for Rough Sea Surface

It is difficult to obtain the complex boundary effects of electromagnetic wave refraction and reflection of the information for a rough sea surface. When the distance is long, the grazing angle is smaller, which make the problem more complicated. For these problems, the method of equivalent reflection coefficient or equivalent surface impedance is often used in engineering applications to consider the roughness of sea surfaces, and it is thus possible to study the propagation of electromagnetic waves on rough sea surfaces by using the parabolic equation.

### 2.1. Parabolic Equation Method

In most cases, PE uses an expression under the Cartesian coordinate system *xyzO*. Assuming that the wave propagates in a small angular direction toward the +*x* direction, then after the PE derived from the two-dimensional scalar Helmholtz equation and Taylor’s expansion takes the first two approximations, the standard parabolic equation shown below is obtained:

(1) $$\frac{\partial u\left(x,z\right)}{\partial x}=\frac{ik}{2}\left[\frac{1}{k^{2}}\frac{\partial^{2}}{\partial z^{2}}+n^{2}\left(x,z\right)-1\right]u\left(x,z\right)$$

where ***u***(*x,z*) represents the wave function of the electric or magnetic field; *k* is the wave number in vacuum; *n* is the atmospheric refractive index.

The SSFT solution is written as Ref. [6]:

(2) $$u\left(x+\Delta x,z\right)=e^{\frac{i\Delta xk\left(n^{2}-1\right)}{2}}F^{-1}\left\{e^{-\frac{i\Delta xp^{2}}{2k}}F\left[u\left(x,z\right)\right]\right\}$$

where Δx is the step size of the step; *p* is the amount of transform in the frequency domain; *F*(□) and *F*^−1^(□) are the Fourier transform and its inverse transform. The term exp [*ik*Δ*x*/2(*n*^2^ − 1)] is the refractive factor, which indicates refractive effect of the medium on the electromagnetic wave, which is called the refractive factor; The term exp (*i*Δ*xp*^2^/2*k*) is the diffraction factor, which indicates the diffraction effect of the obstacle on the electric wave during the electromagnetic wave propagation, which is called the diffraction factor; ***u***(*x*_0_,*z*) is the field distribution.

The initial field of the narrow-angle parabolic equation can be obtained by Fourier transform of the pattern function of the transmitting antenna. The impedance boundary of the rough sea surface can be described by the following impedance boundary conditions:

(3) $${\frac{\partial u\left(x,z\right)}{\partial z}|}_{z=0}+{\alpha u\left(x,z\right)|}_{z=0}=0$$

with:

(4) $$\alpha =ik\sin\theta \frac{1-\Gamma_{\gamma }}{1+\Gamma_{\gamma }}$$

where the effective reflection coefficient is Γ*_γ_* = *ρ*Γ; Γ is the Fresnel reflection coefficient; *ρ* is the equivalent reflection coefficient for a rough surface; *θ* is the grazing incidence angle. The Fresnel reflection coefficient is expressed as:

(5) $$\begin{array}{l} \Gamma_{H}=\frac{\sin\theta -\sqrt{\varepsilon -\cos^{2}\theta }}{\sin\theta +\sqrt{\varepsilon -\cos^{2}\theta }}\\ \Gamma_{V}=\frac{\varepsilon \sin\theta -\sqrt{\varepsilon -\cos^{2}\theta }}{\varepsilon \sin\theta +\sqrt{\varepsilon -\cos^{2}\theta }} \end{array}$$

in which subscripts *H* and *V* represent the horizontal polarization and vertical polarization, respectively; *ε* is the complex dielectric constant of seawater.

The equivalent reflection coefficient for a rough surface *ρ* leads to:

(6) ρ=φ¯φ0=∫−∞+∞exp(2ikςsinθ)P(ς)dς

It can be seen from Equation (6) that the equivalent reflection coefficient depends on the height of the rough surface from the Probability Density Function (PDF), so it is very important to obtain an accurate PDF.

### 2.2. Miller-Brown Method

**Figure 2:**
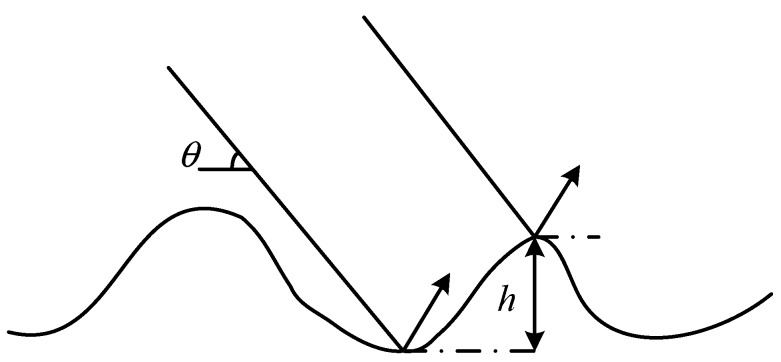
Schematic diagram of Rayleigh roughness parameters. The Rayleigh roughness factor *γ* represents the phase change between two reflecting rays at the grazing angle *θ* and root mean square height difference *h*. When *γ* is small, the reflection line of electromagnetic wave is approximate to the same phase, so the surface can be approximated to a smooth surface.

As shown in Figure 2, if the plane wave irradiates the rough sea surface, the Rayleigh roughness factor can be expressed as:

(7) γ=2khsinθ

where *k* is the wave constant; *θ* is the grazing angle and the root mean square height difference *h* are calculated by the Phillips spectrum:

(8) $$h=0.0051w^{2}$$

where *w* is the sea surface wind speed, in units of m/s.

Assuming that the height of rough sea surface ς(x,y) is a slowly varying function, according to Kirchhoff approximation, the surface is treated as local flattening. Then the reflection field of the average mirror reflection direction is:

(9) $$\bar{\varphi }=\varphi_{0}\int_{-\infty }^{+\infty }\exp\left(2ik\varsigma \sin\theta \right)P\left(\varsigma \right)d\varsigma$$

where $\varphi_{0}$ is a mirror reflection field of a flat plane; P(ς) is a probability density function of the surface height. Miller proposed a more reasonable altitude method with approximate rough sea surface distribution [9]:

(10) $$P_{1}\left(\varsigma \right)=\frac{1}{\pi^{3/2}h}\exp\left(-\frac{\varsigma^{2}}{8h^{2}}\right)K_{0}\left(\frac{\varsigma^{2}}{8h^{2}}\right)$$

where *K*_0_ is the second kind of modified Bessel function. It is concluded that the roughness attenuation factor under this distribution is:

(11) $$\rho_{1}=\exp\left(-\frac{1}{2}\gamma^{2}\right)I_{0}\left(\frac{1}{2}\gamma^{2}\right)$$

where *I*_0_ is the zero-order modified Bessel function.

### 2.3. Shadowing Effect Method

Here the formula for calculating the roughness attenuation factor is:

(12) $$\rho =\int_{-\infty }^{+\infty }\exp\left(-2ik\varsigma \sin\theta \right)P\left(\theta ,\varsigma \right)d\varsigma$$

**Figure 3:**
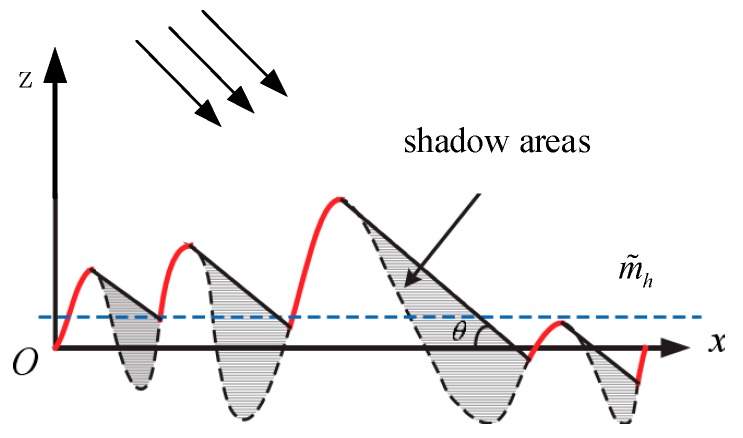
The sketch of the shadowing effect of waves; ${\tilde{m}}_{h}$ is the average wave height. Because of the shadow effect, electromagnetic waves can only illuminate a part of the sea surface, and the roughness attenuation factor is different from Miller-Brown method.

As shown in Figure 3, the probability density function of rough surface height is related to the grazing angle. Fabbro gives the PDF by the Smith method:

(13) $$\tilde{P}\left(\theta ,\;\zeta \right)=P_{\zeta }\left(\zeta \right)\left(1+2\Lambda \right){\left[F\left(\zeta \right)\right]}^{2\Lambda }$$

where $P_{\zeta }\left(\zeta \right)$ is the standard Gauss distribution:

(14) $$\{\begin{array}{c} \Lambda \left(v\right)=\frac{\exp\left(-v^{2}\right)-v\sqrt{\pi }erfc\left(v\right)}{2v\sqrt{\pi }},\;v=\frac{\tan\theta }{\sqrt{2}\sigma_{\gamma }}\\ F\left(\zeta \right)=1-\frac{1}{2}\mathrm{erfc}\left(\zeta /\sqrt{2}\sigma_{\zeta }\right) \end{array}$$

where $\sigma_{\zeta }$ is the standard deviation of height distribution and $\sigma_{\gamma }$ is the standard deviation of slope. Generally, the following formulas $\{\begin{array}{c} \sigma_{\zeta }\approx 6.28\times 10^{-3}u_{10}^{2.02}\\ \sigma_{\gamma }\approx 5.62\times 10^{-2}u_{10}^{0.5} \end{array}$ are used, in which the wind speed is 10 m above sea level [14].

If $h=\zeta /\left(\sqrt{2}\sigma_{\zeta }\right)$ is the normalized height, the mean and variance of the normalized height of the sea surface can be obtained as follows:

(15) $$\{\begin{array}{c} {\tilde{m}}_{h}=\int_{-\infty }^{+\infty }hP\left(h;\theta \right)dh\\ {\tilde{\sigma }}_{h}^{2}=\int_{-\infty }^{+\infty }{\left(h-{\tilde{m}}_{h}\right)}^{2}P\left(h;\theta \right)dh \end{array}$$

By substituting (14) and (15) into (16), the following formula is obtained:

(16) $$\{\begin{array}{c} {\tilde{m}}_{h}\left(v\right)=\frac{1+2\Lambda }{\sqrt{\pi }}\int_{-\infty }^{+\infty }he^{-h^{2}}{\left[1-erfc(h)/2\right]}^{2\Lambda }dh\\ {\tilde{\sigma }}_{h}^{2}\left(v\right)=\frac{1+2\Lambda }{\sqrt{\pi }}\int_{-\infty }^{+\infty }{\left(h-{\tilde{m}}_{h}\right)}^{2}e^{-h^{2}}{\left[1-erfc(h)/2\right]}^{2\Lambda }dh \end{array}$$

Finally, the roughness attenuation factor considering the shadow effect method is obtained as follows:

(17) $$\rho_{2}=\exp\left(-2ik{\tilde{m}}_{h}\sin\theta -{\left(2ik\sin\theta \right)}^{2}/2\right)$$

By synthesizing the attenuation factors of Miller-Brown model and shadow effect model, the influence of shadow effect on the attenuation factor of roughness can be analyzed, which considers not only the attenuation of electromagnetic wave from the rough sea surface, but also the phase effect in the process of electromagnetic wave propagation.

Because a rough sea surface is corrected by the reflection coefficient, we should pay attention to the two following points when using equivalent reflection coefficient of a rough sea surface: when the parabolic equation is used to solve the problem of electromagnetic wave propagation on a rough sea surface, the impedance condition of the lower boundary will change with the appearance of equivalent reflection coefficient, so SSFT must reconsider the impedance condition at each step.

In the case of a rough sea surface, the estimation of grazing angle is no longer a simple case like for a smooth sea surface. Then, we need to use spectral estimation method or some advanced optical methods to estimate the grazing angle [15,16]. MUSIC method is commonly used as the representative of spectral estimation method.

## 3. Random Sea Surface Model for Parabolic Equation

### 3.1. Three-Dimensional Random Sea Surface Double Superposition Model

In the sea surface modeling, spectrum-based statistical methods are often used to describe the sea surface. Many researchers all over the world have proposed a large number of sea wave spectra such as PM spectrum [17], FUNG spectrum [18] and JONSWAP spectrum [19]. JONSWAP (the joint North Sea wave program) derives spectral functions by using measured wind speeds and wind spectrum fitting. Moreover, JONSWAP is characterized by moderate winds and limited wind distances. Most experience shows that it is in good agreement with the measured results. It is applicable to a wide range of applications in wind waves at different growth stages. The JONSWAP spectrum is an internationally accepted international standard ocean spectrum model, but this method needs to be based on a complex mathematical model with a large amount of calculation.

The actual wave motion is a complex three-dimensional random process. The two-dimensional wave simulation cannot effectively reflect the actual wave motion. Therefore, a double superposition model is proposed to accurately simulate three-dimensional random waves. Previous experience shows that this spectrum is in good agreement with the measured results and is applicable to a wide range of applications in wind waves at different growth stages. Under this model, the three-dimensional wave surface equation can be expressed as Ref. [20]:

(18) $$\eta \left(x,y,t\right)=\sum_{i=1}^{m}\sum_{j=1}^{n}\zeta_{ij}\cos\left[k_{{}_{ij}}\left(\left(x+V_{x}t\right)\cos\theta_{j}+\left(y+V_{y}t\right)\sin\theta_{j}\right)-\omega_{ij}t+\beta_{ij}\right]$$

(19) $$\omega_{ij}=\left(\omega_{i}+\omega_{i+1}\right)/2-\frac{1}{2}\Delta \omega +\left(j-1+RAN(i,j)\right)\Delta \omega /n$$

where *m* is the number of frequency division, *n* is the number of directional division, $\zeta_{ij}$, V, $k_{{}_{ij}}$, $\theta_{j}$, $\omega_{ij}$ and $\beta_{ij}$ are the wave amplitude, speed of ships, wave number, direction angle, frequency and phase angle respectively. With $k_{i}=\omega_{di}^{2}/g$. $\beta_{ij}$ represents the random initial phase uniformly distributed in the range of 0–2π, RAN is a random number uniformly distributed in the [0, 1] interval.

The wave amplitude $\zeta_{ij}$ is expressed as the following formula:

(20) $$\zeta_{ij}=\sqrt{2S\left(\omega_{i},\theta_{j}\right)\Delta \omega \Delta \theta }$$

where *S*(*ω_i_*,*θ_j_*) is the direction spectrum function, *ω_i_* denotes the circular frequency of the *n*-th component wave, *θ_j_* is the (*i,j*)-th direction angle, Δ*ω* is the frequency division interval, Δ*θ* is the angular separation interval, and *ω_i_* = *ω_L_* + (*i* − ½)Δ*ω*, *θ_j_* = *θ*_min_ + (*j* − ½)Δ*θ*:

(21) $$\Delta \omega =\frac{\left(\omega_{H}-\omega_{L}\right)}{m},\;\Delta \theta =\frac{\theta_{\max}-\theta_{\min}}{n}$$

where *ω_H_* and *ω_L_* represent the upper and lower limits of the frequency range, the value depends on the required accuracy. *θ*_max_ and *θ*_min_ represent the upper and lower limits of the range of the direction angle. The spectral function *S*(*ω*) satisfies the following formula:

(22) $$S\left(\omega \right)=\alpha_{s}\frac{g^{2}}{\omega^{5}}\exp\left[-\frac{5}{4}{\left(\frac{\omega_{m}}{\omega }\right)}^{4}\right]\gamma^{\exp\left[-{\left(\omega -\omega_{m}\right)}^{2}/\left(2\sigma^{2}\omega_{m}^{2}\right)\right]}$$

where *γ* is peak lift factor, which is between 1.5 and 6, *σ* is a factor of the shape of the wave, *α*_S_ is a factor of wave energy, *ω_m_* is the frequency of a wave crest.

*α*, *σ* and *ω_m_* satisfy the following formula:

(23) $$\{\begin{array}{c} \begin{array}{cc} \sigma =0.07 & \omega \leq \omega_{m} \end{array}\\ \begin{array}{cc} \sigma =0.09 & \omega >\omega_{m} \end{array} \end{array},\;\alpha =0.076{\left(\tilde{X}\right)}^{-0.22},\;\omega_{m}=22\left(g/U_{10}\right){\left(\tilde{X}\right)}^{-0.33}$$

where $\tilde{X}$ is the dimensionless zone length:

(24) $$\tilde{X}=gX/U_{10}^{2}$$

where *X* is the wind distance, *U*_10_ is the wind speed at 10 m above sea level.

Since the JONSWAP spectrum describes the change of energy with frequency, and the energy distribution of three-dimensional random waves is related to frequency and direction angle, and the influence of frequency and direction angle is independent of each other, the directional spread spectrum function *G*(*θ_d_*) is only related to the direction angle *θ_d_*:

(25) $$G\left(\theta_{d}\right)=G_{0}\left(s\right)\cos^{2s}\frac{\theta_{d}-\theta_{0}}{2}$$

where *θ*_0_ is the main direction of the wave; *s* is the concentration parameter of the direction distribution; the coefficient *G*_0_ is determined by the following formula:

(26) $$G_{0}\left(s\right)=\frac{1}{\pi }2^{2s-1}\frac{\Gamma_{1}{}^{2}\left(s+1\right)}{\Gamma_{1}\left(2s+1\right)}$$

where Γ_1_ is the gamma function.

*s* and ω satisfy the relationship:

(27) $$s=\{\begin{array}{c} s_{\max}{\left(\omega /\omega_{m}\right)}^{5}\begin{array}{cc} & \omega \leq \omega_{m} \end{array}\\ s_{\max}{\left(\omega /\omega_{m}\right)}^{-2.5}\begin{array}{cc} & \omega >\omega_{m} \end{array} \end{array}$$

where for the wind into a wave, *s*_max_ generally takes 5–15, with an average of 10.

Therefore, the directional spectrum of three-dimensional random waves *S*(*ω,θ*) can be written as follows:

(28) S(ω,θ)=S(ω)G(θ)

**Figure 4:**
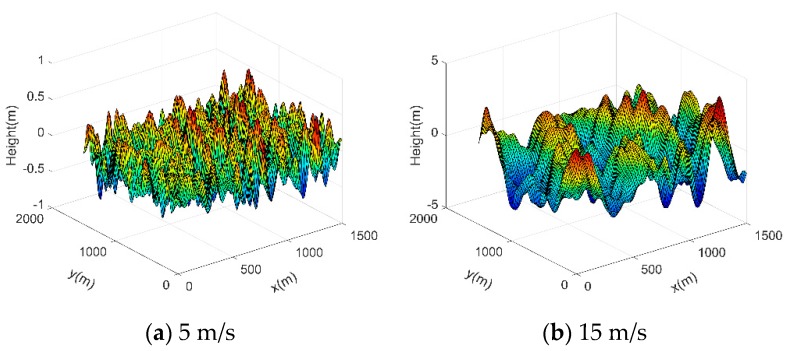
3-D random sea surface with a wind speed of 5 m/s and 15 m/s.

Under the rough sea surface conditions, the main direction of the wave *θ*_0_ is 0°, *V*_x_ = *V_y_* = 0, *t* = 0 and the gravitational acceleration is 9.81 m/s^2^. The accuracy of the random sea surface model has been verified by Ref. [19]. And the model can better meet the real wave conditions. Figure 4 shows that the larger the wind speed, the steeper the waves, the larger the undulating waves.

### 3.2. Piecewise Linear Shift Transform Method

By establishing a three-dimensional random sea surface double superposition model, random sea surface wave height profiles at different wind speeds can be obtained, which can be approximated to complex topographic height profiles. The parabolic equation topographic model is introduced to select the sea surface boundary conditions. Then, the parabolic equation in the treatment of irregular terrain mainly includes terrain shading method, displacement transformation method, conformal transformation method, etc., [21,22,23]. The piecewise linear shift transformation method is the most precise terrain processing method in parabolic equation method. The terrain model and the continuous shift transformation method used in the derivation process are the same. The undetermined variables *θ* are introduced and used as the phase function of the field:

(29) $$\theta \left(x,z\right)=kz^{\prime }T^{\prime }+f\left(x\right)$$

where $K_{0}=\frac{k}{\sqrt{1+T^{\prime 2}}}$; $f\left(x\right)=K_{0}\left(1+T^{\prime }\right)$, the derived Feit-Fleck parabolic equation is:

(30) $$\begin{array}{l} \frac{\partial u\left(x,z\right)}{\partial x}=\frac{ik}{\sqrt{1+T\prime^{2}}}\left(\sqrt{1+\frac{1+T\prime^{2}}{k^{2}}\frac{\partial^{2}}{\partial z\prime^{2}}}-1\right)u\left(x,z\right)+\\ ik\left(\sqrt{n^{2}-\frac{T\prime^{2}}{1+T\prime^{2}}}-1\right)u\left(x,z\right) \end{array}$$

It can be seen that, compared with the Feit-Fleck parabolic equation in flat terrain, the former formula *k* only replaces the original wave constant *k*/1 + *k*^2^, and $\sqrt{n^{2}+T^{\prime 2}/(1+T^{\prime 2})}$ will replace *n*. Therefore, the diffraction factor and refraction factor in flat model will be modified separately.

There are:

(31) T′=tan(β)

(32) $$\{\begin{array}{c} \frac{k}{\sqrt{1+T\prime^{2}}}=k\cos\left(\beta \right)\\ \frac{T\prime^{2}}{1+T\prime^{2}}=\sin\left(\beta \right) \end{array}$$

The transformed parabolic equation can still be solved by SSFT, except that the diffraction factor and refraction factor are corrected by the slope of the piecewise terrain. The final SSFT solution is:

(33) $$u\left(x+\Delta x,z\right)=e^{ik\Delta x\left(\sqrt{n^{2}-\sin\beta }-2\right)}F^{-1}\left\{e^{i\Delta x\left(\sqrt{k^{2}\cos\beta -p^{2}}\right)}F\left[u\left(x_{0},z\right)\right]\right\}$$

where the refractive factor $\sqrt{n^{2}-\sin^{2}\beta }$ is corrected, and the diffraction factor $\sqrt{k^{2}\cos^{2}\beta -p^{2}}$ is corrected.

**Figure 5:**
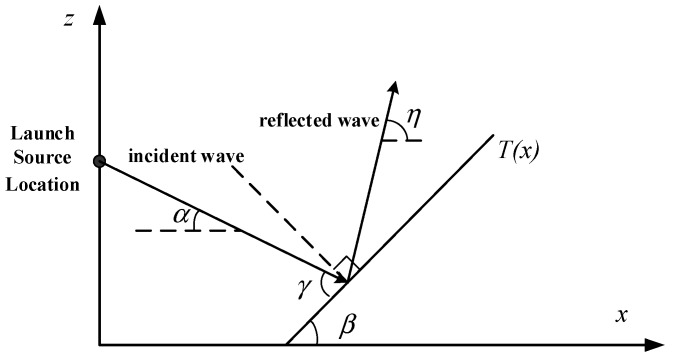
Piecewise linear transformation of terrain. For piecewise linear terrain, assuming the angle *β* between the terrain and the horizontal plane.

When using piecewise linear translation method, the Leontovich impedance boundary conditions need to be revised, and the revised boundary conditions can be obtained as follows:

(34) $$\alpha^{\prime }=\cos\left(\beta \right)\left[\alpha +\tan\beta (1-cos\theta )\right]$$

where *α* reflects the impedance characteristics of the lower boundary and *θ* is the grazing angle of electromagnetic wave which is the angle *γ* in Figure 5.

After that, the irregular sea surface processing is completed. Combined with the SSFT method, the distribution of the radio wave propagation field under random sea surface can be calculated, and then the propagation characteristics of electromagnetic waves in a complex marine environment can be analyzed.

## 4. Parabolic Equation in Sea Atmospheric Dust

When the atmospheric dust meets certain conditions, the trajectory of electromagnetic wave will bend to the Earth’s surface, and the electromagnetic wave will be “captured” by the dust layer. Then over-the-horizon propagation, which is one of the main factors of electromagnetic wave propagation in the sea environment which is different from the propagation in the terrestrial environment.

**Figure 6:**
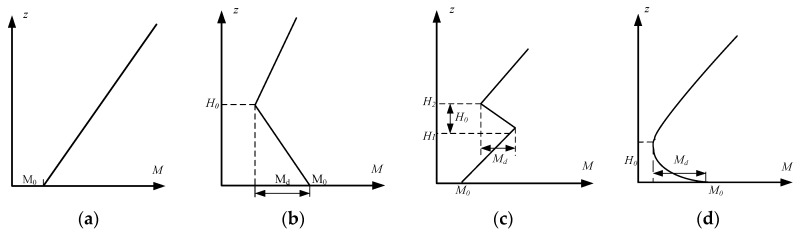
Refractive index profiles of several common atmospheric dust types: (**a**) is the refractive index profile of the standard atmosphere; (**b**) is the refractive index profile of surface dust; (**c**) is the refractive index profile of suspended dust; and (**d**) is the refractive index profile of evaporation dust.

As can be seen from Figure 6, the refractive index is one of the basic conditions for the formation of atmospheric dust and the height shows a negative growth trend. *M* is the corrected atmospheric refractive index, *z* is the height, *H*_0_ is the dust height, *M_d_* is the dust strength, *M*_0_ is the bottom corrected refractive index.

For the dust refractive index profile of evaporation dust, the following formulas are generally used [24]:

(35) $$M\left(z\right)=M\left(z_{0}\right)+c_{0}\left(z-H_{0}\ln\frac{z}{z_{0}}\right)$$

where *M*(*z*_0_) is the bottom modified refractive index (usually *M*(*z*_0_) = 330); *z*_0_ is the roughness length, which usually take *z*_0_ = 1.5 × 10^−4^. *c*_0_ is a constant, which usually takes the value 0.125. For surface dust and suspended dust, bilinear and trilinear profiles can be used to represent them [25]:

(36) $$M\left(z\right)=M_{0}+\{\begin{array}{cc} \begin{array}{c} c_{1}z\\ c_{1}z_{1}+c_{2}\left(z-z_{1}\right)\\ c_{1}z_{1}+c_{2}\left(z_{2}-z_{1}\right)+c_{3}\left(z-z_{3}\right) \end{array} & \begin{array}{c} z<z_{1}\\ z_{1}<z<z_{2}\\ z_{2}\leq z<z_{3} \end{array} \end{array}$$

where *c*_1_~*c*_3_ usually takes the value 0.118.

More complex refractive index profiles of atmospheric dust can be constructed by the combination of the above two methods. In addition to the requirement of atmospheric refractive index, dust propagation of electromagnetic waves has certain requirements for antenna elevation, electromagnetic wave frequency and antenna height.

## 5. Simulation and Analysis

### 5.1. Effect of Atmospheric Dust on Electromagnetic Wave Propagation

The main factors affecting the propagation of electromagnetic waves on the sea surface are discussed, and the propagation characteristics of different atmospheric dust are simulated and analyzed by using parabolic equation, but the premise of the analysis is a smooth sea surface.

The simulation conditions of atmospheric dust are shown in Table 1. The sensors used in this simulation are ship-borne radar and radar signal receiving devices. The methods involved are suitable for general sea conditions. 

Here the sea surface is considered to be flat, and we simulate the influence of atmospheric dust on the propagation of electromagnetic waves on the sea surface by using the Miller-Brown model. The wind speed is 7 m/s. The polarization mode of antenna is horizontal polarization. The simulation results in different atmospheric environments are as follows.

**Figure 7:**
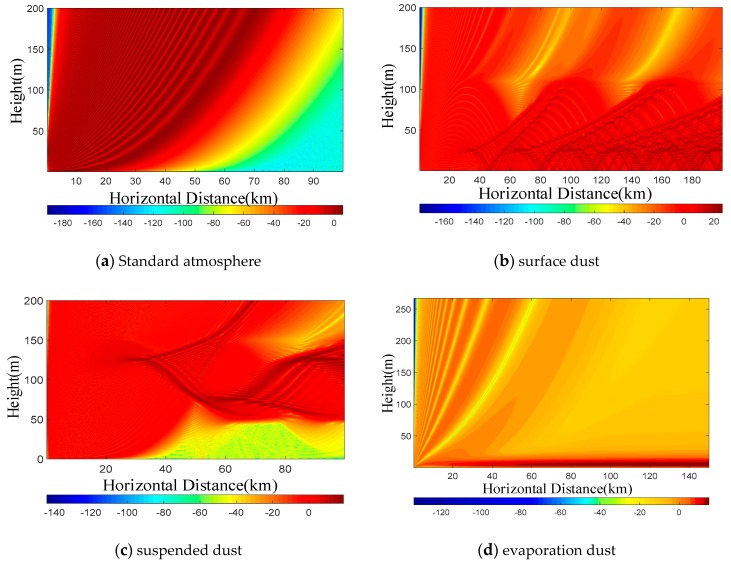
Propagation factors in different atmospheric environments: (**a**) shows the distribution of propagation factors under standard atmosphere; (**b**) is the distribution of propagation factors under surface dust conditions; (**c**) is the distribution of propagation factors under suspended dust conditions; (**d**) is the distribution of propagation factors under evaporation dust conditions.

It can be seen from Figure 7a that the atmospheric refractive index increases affine, so there is no basic condition for the formation of atmospheric dust. Therefore, the distribution of propagation factors in Figure 7a decreases with height. Due to the influence of the atmospheric refractive index, the electromagnetic wave propagates beyond the horizon. In Figure 7b, the propagation factor below the atmospheric dust layer height *H*_0_ = 100 m does not decrease with the height law, but it appears an anomaly larger than that outside the dust layer. Within the horizontal distance of 20 km, there is a little difference between the propagation characteristics and the standard atmosphere. However, the electromagnetic wave “traps” and enters the atmospheric dust layer after 20 km, and the relationship between the attenuation and height of the electromagnetic wave in the atmospheric dust layer is not obvious. Although the electromagnetic wave propagation is abnormal, there is a basic rule: the propagation attenuation in the atmospheric dust layer is smaller than that in other regions. Figure 7c is the distribution of propagation factors under suspended dust conditions. Unlike surface dust, the atmospheric dust layer of suspended dust does not intersect with the lower boundary, but is suspended in the air. Therefore, it can be seen from Figure 7c that the abnormal part of electromagnetic wave propagation is between *H*_1_ ~ *H*_2_. Figure 7d is the distribution of propagation factors under evaporation dust conditions with a dust height of 20 m. The trapping condition is satisfied in the atmospheric dust layer, so the electromagnetic wave propagates beyond the horizon. Compared with the standard atmosphere, the propagation factor of evaporation dust in its atmospheric dust layer is obviously larger, that is, the propagation loss is smaller.

The influence of atmospheric dust on radar electromagnetic wave propagation depends on the radar configuration related to atmospheric dust (e.g., launcher height, type of atmospheric dust, height of atmospheric dust, position of launcher in it, etc.). The phenomenon of atmospheric dust will bring difficulties to predict the electromagnetic wave propagation. Therefore, considering the influence of atmospheric dust on radar coverage, we can change the radar configuration used or avoid the influence of atmospheric dust.

### 5.2. Effect of Rough Sea Surface on Electromagnetic Wave Propagation

When there are waves, the sea surface will become rough, and the electromagnetic waves propagation on the sea surface may have a shadow effect. Therefore, another factor affecting the propagation characteristics of electromagnetic waves is discussed, and the parabolic equation modeling and the simulation under the rough sea surface conditions are also discussed.

The JONSWAP spectrum can’t simulate the shallow water spectrum with more sub-peaks. The piecewise linear shift transformation method can be used to calculate general complex terrains, but its effect on edge peaks is not good, so the model is suitable for non-extreme sea conditions far from the coast, and when the radar antenna is higher than the height of waves. The selection of sensors is related to sea condition and frequency, and the higher the frequency, the greater the influence of radar wave propagation on the rough sea surface.

**Figure 8:**
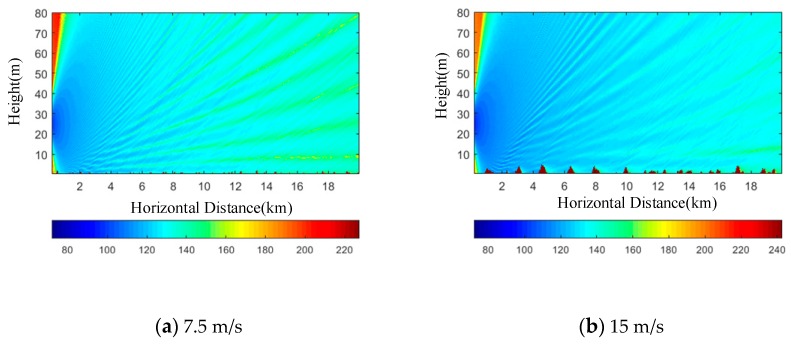
The pseudo-color map of propagation loss distribution. It gives propagation loss pseudo-colour map of the vertical profile when the sea surface wind speed is: (**a**) 7.5 m/s and (**b**) 15 m/s. The evaporation dust has a dust height of 20 m. The signal frequency is 8 GHz.

From Figure 8, it can be seen that the blind area can be visible due to the scattering of electromagnetic waves on a rough sea surface; on the other hand, due to the same phenomenon, the radar range can be reduced. Comparing Figure 8a with Figure 8b, we notice that there are two regions. The first is between 0 and 4 km. In this region, the difference between the two results is no more than 5 dB. This is due to the fact that the concentration of waves propagates directly from the source and is not affected by the sea surface (there is no reflection from the sea surface). The distance in this region is mainly positively correlated with the antenna height. In the second area, there is a more significant difference between the propagation loss under different wind speeds (the degree of jitter is different) in the propagation area (4–20 km). This is due to the influence of sea surface roughness (the greater the wind speed is, the rougher the sea surface is) on the propagation of radio waves (reflected waves), resulting in scattering and shadow effects. Affected by the shadow effect (the larger the propagation distance is, the smaller the incident angle is, and the more serious the shadow effect is), the over-the-horizon phenomenon produced by the evaporation dust is greatly weakened.

What’s more, the simulation results show that the roughness of the sea surface has an effect on the propagation loss: With the increase of wind speed, the scattering and shadowing effects of the electromagnetic wave generated by the sea waves are significantly enhanced, while the interference phenomena caused by the direct wave and the reflected waves on the sea surface are significantly weakened. It is necessary to consider the electromagnetic scattering and shadow effect caused by the roughness and randomness of the sea surface when the prediction model of radio wave propagation is constructed in the marine environment.

#### 5.2.1. Effect of Sea Surface Roughness on Electromagnetic Wave Propagation

The electromagnetic wave propagation characteristics under different roughness conditions are analyzed. The Miller-Brown model is adopted. The experimental conditions are basically the same as given in Table 1, but the wind speed and signal frequency are variable. The propagation characteristics of electromagnetic wave are analyzed from the point of view of propagation loss and attenuation.

**Figure 9:**
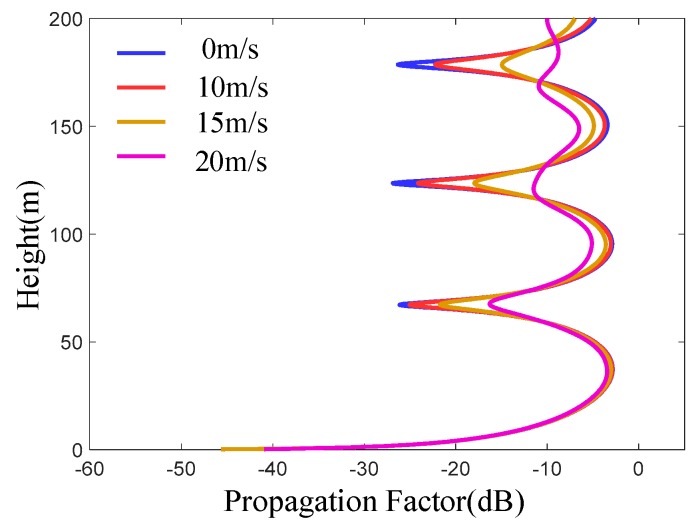
The curve of propagation factor varying with altitude at different roughness of sea surface at 13 km horizontal distance.

From Figure 9, it can be seen that the coherence of electromagnetic waves decreases gradually from windless to 20 m/s wind speed conditions. The coherence decreases with the increase of height. As the wind speed increases, the roughness of the sea surface increases, and the diffuse reflection of electromagnetic wave increases, while the mirror reflection decreases, the coherence of electromagnetic wave decreases. With the increase of height, the grazing angle of electromagnetic wave also increases. Moreover, the roughness factor will gradually increase, so the coherence of electromagnetic wave will gradually weaken. The above two points are consistent with the theoretical results.

**Figure 10:**
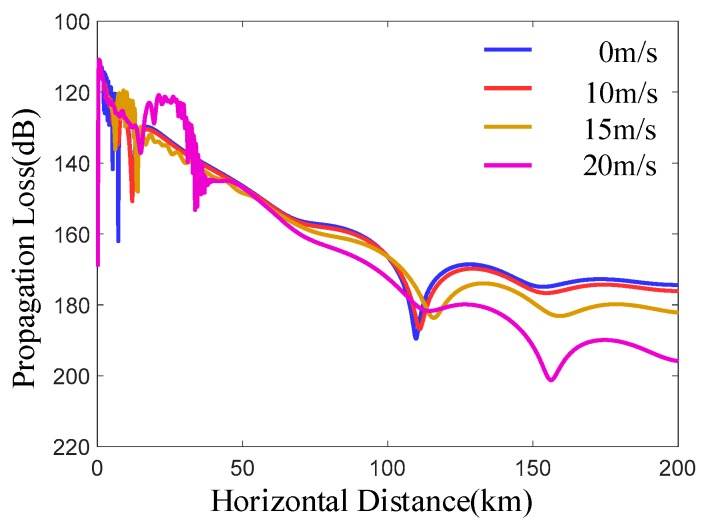
The propagation loss at different sea surface roughness levels. Under different wind speeds, the propagation loss varies with distance, and the atmospheric environment is an evaporation dust. The evaporation dust has a dust height of 20 m, the signal frequency of 8 GHz, an antenna height of 25 m and a receiving height of 15 m.

From Figure 10, it can be seen that the overall transmission loss increases with the horizontal distance, and the corresponding transmission loss increases with the increase of wind speed. This is because when the wind speed increases, the corresponding roughness will also increase. When electromagnetic wave propagates, it will scatter more energy, so the propagation loss will increase.

#### 5.2.2. Considering the Influence of Rough Sea Surface Randomness on Electromagnetic Wave Propagation

After analyzing the influence of different sea surface roughness on electromagnetic wave propagation, the influence of rough sea surface randomness on electromagnetic wave propagation is further discussed. The following simulated atmospheric conditions are standard atmospheres. 

**Figure 11:**
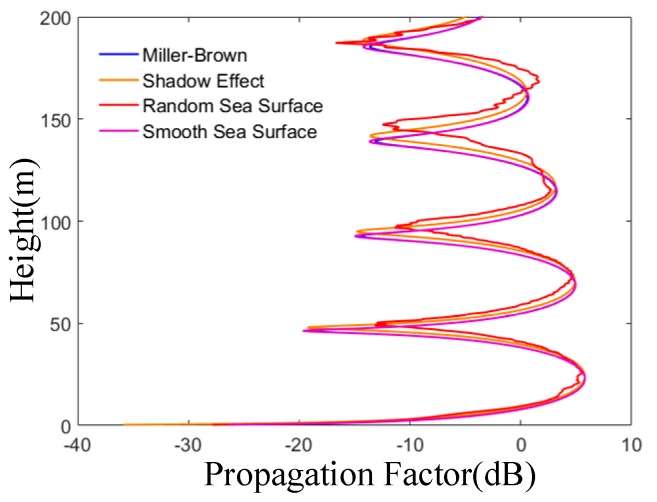
The effects of rough sea surface random on propagation factor. It shows the comparison of propagation factors between a smooth sea surface and a rough sea surface. The roughness attenuation factor of rough sea surface can be divided into Miller-Brown model, shadow effect and random sea surface. The frequency is 1.2 GHz and the wind speed is 7 m/s.

From Figure 11, it can be seen that the rough sea surface considered by the Miller-Brown model only reduces the coherence of electromagnetic waves, which is similar to the results shown in Figure 9. Compared with the Miller-Brown model, the peak height of propagation factor of rough sea surfaces considering the shadow effect is higher, which corresponds to the average increase of wave height of the rough sea surface shown in Figure 3.

Considering the shadow effect, the fluctuation of transmission factors increases, which means that the dynamic range increases. Compared with the shadow effect model, the peak height of propagation factor of the rough sea surface of the random sea surface model is higher, and the propagation factor curve fluctuates to varying degrees. The reason is that the diffuse reflection and shadow effect caused by the geometric characteristics of the random sea surface on electromagnetic waves.

In the three existing models, considering shadow effect increases the accuracy of electromagnetic wave propagation prediction compared with Miller-Brown model, but with the step-by-step solution of the parabolic equation, infinite integrals shown in formula (13) are required in each step, which will also increase the amount of calculation. The random sea surface model based on the parabolic equation takes into account the scattering and shadow effects of electromagnetic waves under rough sea surface conditions, and the model is closer to the actual sea surface conditions, but it needs to generate random sea surface in a certain condition. The random sea surface is calculated as real terrain by piecewise linear shift transformation of the parabolic equation, so the calculation amount is the largest in the three models. Therefore, after discussing the effects of Miller-Brown model, shadow effect and random sea surface model on the propagation factor and loss of electromagnetic wave, we continue to discuss the application scope of each model.

**Figure 12:**
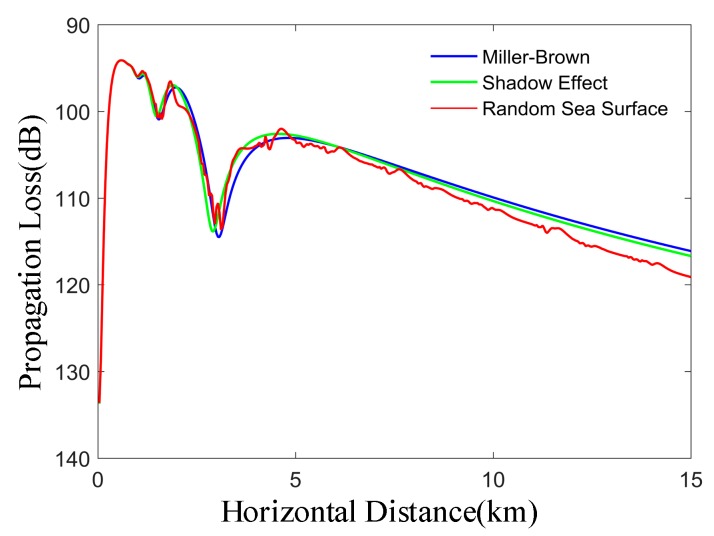
The effects of three models on propagation loss. The roughness attenuation factor of rough sea surface is divided into random sea surface model, Miller-Brown model and propagation loss considering shadow effect.

As shown in Figure 12, the roughness attenuation factor of the rough sea surface is divided into random sea surface model, Miller-Brown model and propagation loss considering shadow effect. Referring to Figure 1, the receiving height is 15 m. It can be seen that the propagation losses of the random sea surface model, Miller-Brown method and considering the shadow effect are a little different at a certain distance (0–10 km), which proves the correctness of the random sea surface model. However, the propagation loss curve of the random sea surface model fluctuates to a certain extent, and the difference will be further exacerbated with the increase of the propagation distance of the electromagnetic wave, especially when the horizontal distance is far from 10 km. Therefore, the rough sea surface generated by the random sea surface model has little influence on the propagation loss of electromagnetic waves for short distances (0–10 km), but the random sea surface increases the propagation loss of microwave waves after 10 km.

The spray zone above the sea, especially in rough sea, has a poor impact on the propagation of microwaves, which is caused by the shadow effect and the electromagnetic scattering phenomenon. Shadow effects are caused when the incident angle is very small and the electromagnetic wave can only irradiate from part of the surface of the wave. Therefore, its causes are first related to the wave height, the transmitting antenna height and the grazing angle. When the wave height is higher, the transmitting antenna height is lower and the grazing angle is smaller, the shadow effect will occur more easily. Secondly, when the wavelength of electromagnetic wave is longer, it will diffract between waves, which will weaken the shadow effect. It means the shorter the wavelength and the higher the frequency of electromagnetic wave, the easier the shadow effect will occur. With the increase of sea surface wind speed, the sea surface becomes rougher, and the electromagnetic scattering phenomenon and shadow effect become more obvious because of the randomness and time-varying characteristics of rough sea surface. Different wind directions will also affect the direction of wave propagation, which will affect the height distribution of random sea surfaces, and then affect the electromagnetic propagation of the sea surface. The following is the simulation and analysis of several situations.

**Figure 13:**
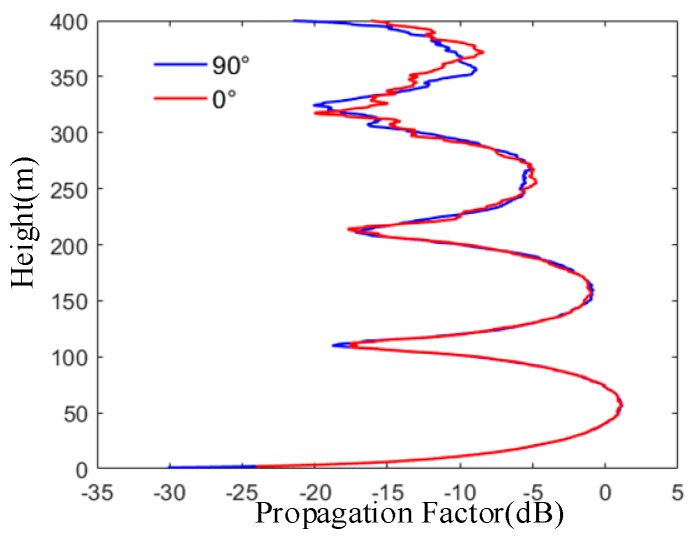
The propagation factor in different wind directions. The variation of propagation factor with altitude is given when the wind speed is 7 m/s and the wind direction is different.

From Figure 13, it can be seen that the higher the altitude is, the more obvious the numerical difference of the propagating factors corresponding to different wind directions is. It will have a certain impact on the propagation characteristics of electromagnetic waves. Therefore, it is necessary to consider the wind direction in calculating the propagation characteristics of radio waves with higher altitude above the sea surface. The existing Miller-Brown model and the shadow effect cannot consider the sea surface wind direction, so the random sea surface model built is reliable.

**Figure 14:**
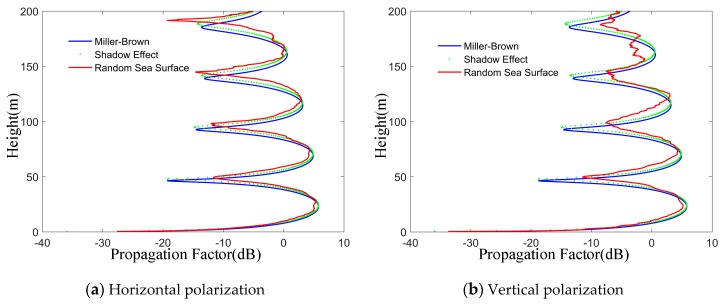
The effects of polarization on different models. The two examples (**a**) Horizontal polarization and (**b**) Vertical polarization, show propagation factors of random sea surface model, Miller-Brown model and shadow effect at different polarization modes. The wind speed is 7 m/s. The propagation distance is 13 km.

As can be seen from the Figure 14, the propagation factors of Miller-Brown model and shadow effect model are almost unchanged and close to each other, regardless of whether the antenna is vertically polarized or horizontally polarized. At the same time, compared with the model considering shadow effect, the propagation factor of the random sea surface model has a greater degree of curve jitter at higher receiving altitude. However, compared with horizontal polarization, at a certain altitude, the shading effect of stochastic sea surface model begins to show obvious differences, and the shaking degree of the curve increases. When the antenna is vertically polarized, the sea surface scattering has a great influence on the propagation of electromagnetic waves. In other words, vertical polarization will make the propagation loss of radar wave on rough sea surface larger. Therefore, when the antenna is horizontally polarized, the shadow effect model can effectively reduce the computational complexity.

**Figure 15:**
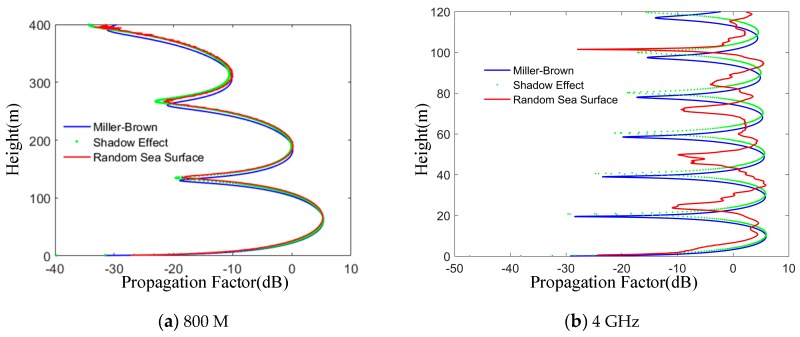
The frequency effects on different methods. The two examples (**a**) 800 M and (**b**) 4 GHz, show propagation factors of random sea surface model, Miller-Brown model and shadow effect at different frequencies.

The propagation distance is 13 km, and the frequencies are 800 MHz and 4 GHz, respectively. As can be seen from the Figure 15, compared with 4 GHz, the propagation factors of the three models are close when the frequencies are lower than 800 MHz. However, due to the diffuse reflection of the rough sea surface, the propagation factor curves of the stochastic sea surface model at certain altitudes fluctuate to varying degrees. Therefore, the influence of sea surface scattering and shadow effect on electromagnetic wave propagation is small when the frequency is small, the wavelength is long and the height is low. 

**Figure 16:**
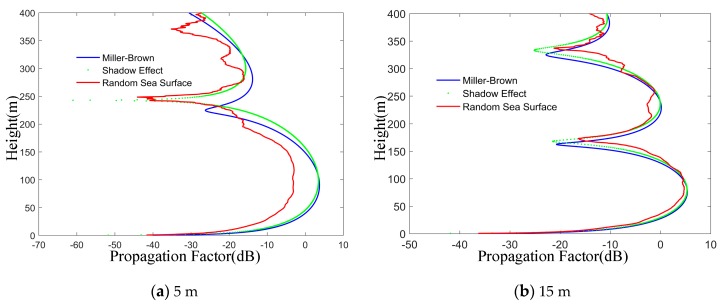
The effects of antenna height on three methods. The two examples; the antenna height (**a**) 5 m and (**b**) 15 m, show comparison of the propagation factors on random sea surface model, Miller-Brown model and shadow effect at different altitudes of the transmitting antenna.

It can be seen from Figure 16, the propagation factors of Miller-Brown model and shadow effect model are close to each other when the antenna height is 15 m compared with the antenna height of 5 m. Therefore, when the transmitting antenna is high and the grazing angle is large, the shadow effect has little influence on the electromagnetic wave. At the same time, compared with the model considering the shadow effect, the propagation factor of the random sea surface model is smaller when the antenna height is higher. The grazing angle is inversely proportional to the propagation distance in the process of electromagnetic wave propagation. According to the above simulation analysis, when calculating the propagation characteristics of electromagnetic wave on rough sea surface, the influence of sea surface scattering and shadow effect can be considered at a long distance to reduce the calculation amount.

**Figure 17:**
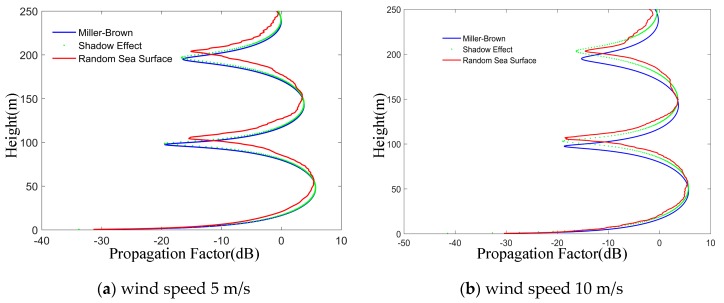
Effects of sea surface wind speed by three methods. (**a**,**b**) are the wind speed 5 m/s and 10 m/s, show a comparison of the propagation factors on Miller-Brown model, shadow effect model and random sea surface model at different wind speeds.

From Figure 17, it can be seen that the propagation factors of the Miller-Brown model and shadow effect model vary smoothly with height, and their peak values decrease gradually with the increase of wind speed. Compared with the sea surface wind speed of 10 m/s, the propagation factors of Miller-Brown model and shadow effect model are close at 5 m/s wind speed, while the shadow effect has less influence on electromagnetic wave at lower wind speed. At a certain altitude, with the increase of wind speed, the shadowing effect of random sea surface model begins to show obvious differences, and the degree of curve jitter increases. When the sea surface wind speed is large (the wave height is higher), the scattering and shadowing effects of the sea surface have a greater impact on the propagation of electromagnetic waves. Therefore, rough sea reduce the range of electromagnetic wave propagation. Moreover, the shadow effect model can effectively reduce the amount of calculation when the sea surface wind speed is small.

Combined with the above simulation, for the transmitter with high antenna height and low frequency, when the sea surface wind speed is small, it is desirable to use shadow effect or Miller-Brown model to calculate the sea surface space. Moreover, the influence of rough sea surface on radar electromagnetic wave propagation depends on the radar configuration (e.g., transmitter height, frequency, polarization mode, transmitter location etc.) and the roughness of sea surface (wind speed and direction affect the roughness of the sea surface). Rough sea surfaces will bring difficulties to the prediction of electromagnetic wave propagation. In fact, we can change the radar configuration to utilize or avoid the influence of rough sea surface on radar wave propagation.

## 6. Conclusions

Prediction and research of electromagnetic wave propagation characteristics in marine environments can provide important reference values for practical engineering applications such as design of marine communication links and the evaluation of radar detection performance. The prediction of electromagnetic wave propagation in marine environments is mainly influenced by the characteristics of the propagation domain (ocean atmosphere, atmospheric dust, etc.) and the reflection effect of sea surface (smooth or rough surface). Therefore, the propagation characteristics of radar electromagnetic wave in several atmospheric dust (the sea surface is considered flat) are simulated and analyzed, and the propagation of electromagnetic wave in dust layer under different atmospheric dust conditions is obtained. Then, a three-dimensional random sea surface model of parabolic equation is introduced to describe the rough sea surface in detail because Miller-Brown model and shadow effect cannot fully consider the electromagnetic scattering of ocean waves, and the influence of the rough sea surface on electromagnetic wave propagation is simulated by the parabolic equation. In this paper, a new method is proposed to simulate the effect of sea surface roughness. The method uses the JONSWAP spectrum to generate three-dimensional sea surface with different wind speeds, simulates the real sea surface to the greatest extent, and uses the parabolic equation to simulate the effect of sea surface roughness on electromagnetic wave propagation. By comparing the Miller-Brown model, shadow effect and stochastic sea surface model under different sea surface wind speeds, frequency, antenna heights, antenna polarization modes and other factors, it is shown that the method can better reflect the influence of wave geometric characteristics on electromagnetic wave propagation. Then the effects of different radar configurations (frequency, antenna height, polarization mode) and sea surface conditions (rough conditions caused by sea surface wind speed) on radar radio wave propagation are obtained. This has important reference value for predicting the wave propagation on the sea surface, and has certain guiding significance for the detection and communication of marine radar systems.

The method presented in this paper offers an original approach to the introduction of sea surface roughness in electromagnetic wave propagation modelling. However, in order to better quantify the contribution of this method and measure the roughness improvement introduced in electromagnetic wave propagation modeling, we plan to do further research that we will soon carry out. First, we will compare the results of different spectra. If possible, other comparisons can be made with real data.

## Figures and Tables

**Table 1:** The simulation conditions of atmospheric dust.

Antenna	Frequency/GHz	Boundary Condition	Antenna Height/m	Atmospheric Environment
Gauss	20	Sea surface	25	Common atmospheric dust
